# Influence of afatinib dose on outcomes of advanced EGFR-mutant NSCLC patients with brain metastases

**DOI:** 10.1186/s12885-018-5110-2

**Published:** 2018-12-03

**Authors:** Wan-Ling Tan, Quan Sing Ng, Cindy Lim, Eng Huat Tan, Chee Keong Toh, Mei-Kim Ang, Ravindran Kanesvaran, Amit Jain, Daniel S. W. Tan, Darren Wan-Teck Lim

**Affiliations:** 1 Division of Medical Oncology, National Cancer Centre Singapore, Singapore, Singapore; 2 Clinical Trials & Epidemiological Sciences, National Cancer Centre Singapore, Singapore, Singapore; 3 Genome Institute of Singapore, A*STAR, Singapore, Singapore; 4 Institute of Molecular and Cell Biology, A*STAR, Singapore, Singapore

**Keywords:** EGFR mutation NSCLC, Metastatic, Afatinib, Brain metastases, Dose

## Abstract

**Background:**

Afatinib is an oral irreversible epidermal growth factor receptor (EGFR) tyrosine-kinase inhibitor (TKI) indicated in first-line treatment of advanced EGFR-mutant (EGFRm+) non-small cell lung cancer (NSCLC). Dose dependent side effects can limit drug exposure, which may impact on extracranial and central nervous system (CNS) disease control.

**Methods:**

We performed a retrospective study of 125 patients diagnosed with advanced EGFRm+ NSCLC treated with first-line afatinib at a tertiary Asian cancer center, exploring clinicopathological factors that may influence survival outcomes. Median progression free survival (PFS) was estimated using the Kaplan-Meier method. Comparison of PFS between subgroups of patients was done using log-rank tests and Cox proportional hazards models.

**Results:**

Out of 125 patients, 62 (49.6%) started on 40 mg once daily (OD) afatinib, 61 (48.8%) on 30 mg OD and 1 (0.8%) on 20 mg OD. After median follow-up of 13.8 months from afatinib initiation, the observed response rate was 70.4% and median PFS 11.9 months (95% CI 10.3–19.3). 42 (33.6%) patients had baseline brain metastases (BM) and PFS of those who started on 40 mg OD (*n* = 17) vs. 30 mg OD (*n* = 25) was 13.3 months vs. 5.3 months (HR 0.39, 95% CI 0.15–0.99). BM+ patients who started on 40 mg had similar PFS to patients with no BM (13.3 months vs. 15.0 months; HR 0.79, 95% CI 0.34–1.80).

**Conclusion:**

In patients with advanced EGFRm+ NSCLC with BM+, initiating patients on afatinib 40 mg OD was associated with improved PFS compared to 30 mg OD, underscoring the potential importance of dose intensity in control of CNS disease.

## Background

Epidermal growth factor receptor (EGFR) tyrosine kinase inhibitors (TKIs) are the standard of care for first-line treatment of advanced NSCLC with sensitizing EGFR mutations [1, 2]. Despite high response rates of 60–70%, treatment failure inevitably ensues after a median duration of 10–18 months, regardless of choice of TKI. Although the emergence of genomic alterations commonly accounts for secondary resistance to EGFR TKI, CNS failure is often attributed to inadequate penetration into the CNS – regarded as a ‘sanctuary’ site. Indeed, the lifetime risk of brain metastases (BM) is more than 30% of patients in EGFR mutant NSCLC, and where present, has traditionally been associated with poorer survival [3–5].

Although intracranial efficacy of first-line EGFR TKIs has not been established in prospective large-scale studies, clinical observations from trials support intracranial activity with afatinib – a second-generation, irreversible pan-human epidermal growth factor receptor (HER) inhibitor. In a combined post-hoc analysis on patients with asymptomatic baseline BM from the LUX-lung 3 and LUX-lung 6 studies, afatinib significantly improved the objective response rate (RR) and progression-free survival (PFS) compared to chemotherapy [3, 6]. However, due to potent EGFR wild-type inhibition, afatinib is associated with increased skin and gastrointestinal toxicities, resulting in dose reductions reported in up to 53.3 and 28% patients in the randomized LUX-Lung 3 and 6 trials respectively [7]. The potential impact of dose reductions with afatinib on CNS disease control also remains poorly characterized.

We performed a retrospective study to evaluate the clinicopathological factors affecting survival outcomes of patients with EGFRm+ NSCLC treated with first-line afatinib, specifically examining the impact of starting dose in patients with or without BM at diagnosis.

## Methods

### Study population

We retrospectively analyzed 125 consecutive patients with advanced/stage IV EGFRm+ NSCLC treated with first-line afatinib between January 2012 to February 2017 at the National Cancer Centre Singapore (NCCS) and consented to data collection for research purposes. We included eligible patients under our Lung Cancer Consortium Singapore (LCCS) data-base up to February 2017. Patients were analyzed for RR and PFS as per investigator-assessed Response Evaluation Criteria in Solid Tumors (RECIST 1.1) criteria. After initiation of afatinib, radiological assessments of patients were performed at 2–3 month intervals as decided by the treating physician, with brain imaging by either contrasted computed tomography (CT) or magnetic resonance imaging (MRI) brain performed regularly for patients with documented brain metastases. An exploratory analysis was done for clinical factors that influenced survival. Reflex EGFR mutational analysis was performed by direct Sanger sequencing or Roche COBAS EGFR mutation test v2 [8–11]. This research was approved by our local Centralized Institutional Review Board (CIRB) and data was collected and subsequently analyzed anonymously prior to reporting.

### Statistical analysis

PFS was defined as time from start of afatinib treatment to progression or death. Median PFS was estimated using the Kaplan-Meier method. Comparison of PFS between subgroups of patients was done using log-rank tests and Cox proportional hazards models. Two-sided *p*-values less than 0.05 were considered statistically significant. All analyses were performed in Stata (Version 14.2, StataCorp, Texas, USA).

## Results

### Clinico-pathologic characteristics

The baseline characteristics of the 125 patients with EGFRm+ lung cancer who received first-line afatinib are summarized in Table 1. The median age at diagnosis was 62 years (range 26–86) and 121 (96.8%) had adenocarcinoma. 87 (69.6%) patients had EGFR exon 19 deletion, 27 (21.6%) had L858R mutation, and the rest (8.8%) had other EGFR mutations including G719A, E697Q, exon 20 mutations like A763_Y764insFQEA, double mutations or unknown. 95 (76.0%) patients were never-smokers and the remaining were former/current smokers. Of note, 42 (33.6%) patients had BM prior to afatinib initiation. 62 (49.6%) started on 40 mg once daily (OD) afatinib, 61 (48.8%) on 30 mg OD and 1 (0.8%) on 20 mg OD at the treating physician’s discretion, due to concerns about drug tolerability.

**Table 1:** Patient Baseline Characteristics. The baseline demographics and clinical characteristics of patients with advanced EGFRm+ NSCLC treated with first-line afatinib (*n* = 125) in our cohort

Characteristic	No.	%
Sex		
Male	64	51.2
Female	61	48.8
Age at diagnosis, years		
Median	62	
Range	26–86	
Ethnicity		
Chinese	100	80.0
Malay	14	11.2
Indian	3	2.4
Others	8	6.4
Smoking status		
Never	95	76.0
Former	17	13.6
Current	13	10.4
Histotype – NSCLC		
Adenocarcinoma	121	96.8
Adenosquamous carcinoma	1	0.8
NOS	3	2.4
EGFR mutation type		
Exon 19 deletion^[a]^	87	69.6
Exon 21 L858R	27	21.6
Others^[b]^	11	8.8
Brain metastases at baseline		
No	82	65.6
Yes	42	33.6
Unknown	1	0.8
Starting dose of afatinib once daily (OD)		
40 mg	62	49.6
0 mg	61	48.8
0 mg	1	0.8
Unknown	1	0.8

^[a]^E746_A750del; E746_A750delinsIP; E746_A750delinsQP; E746_A750delinsVP; E746_T751delinsV; E746_S752delinsV; E746_P753delinsVS; L747_A750delinsP; L747_T751del; L747_P753delinsS; NOS

^[b]^E697Q; A763_Y764insFQEA; Double mutation; Unknown

*NSCLC* Non-small cell lung cancer, *NOS* Not otherwise specified

### Factors influencing outcomes to afatinib

Median follow-up time was 13.8 months (95% CI 11.5 to 19.5 months) from start of afatinib treatment. Median duration of afatinib treatment was 8.7 months. At the time of data analysis in February 2017, 52 patients (41.6%) were still on afatinib. RR with afatinib was 70.4% and the disease control rate was 77.6%. No complete response (CR) was seen, while 11.2% had progressive disease (PD) as best overall RECIST response. The median PFS was 11.9 months (95% CI 10.3 to 19.3 months). Table 2 summarizes the clinical factors influencing PFS outcomes to afatinib in the total population by univariate analysis. Smoking history and EGFR mutation type were statistically significant clinical factors associated with PFS (log-rank *p* = 0.017 and < 0.001, respectively). Interestingly, in patients with brain metastases, a lower starting dose was found to have a detrimental effect on outcomes.

**Table 2:** Factors influencing outcomes to afatinib. The clinical factors that influenced PFS in our cohort

	No. of events/ patients	Median PFS, months (95% CI)	Log-rank *p*-value	Hazard ratio (95% CI)	Cox model *p*-value
Total	60 / 120	11.9 (10.3, 19.3)	NA	NA	NA
Sex					
Male	35 / 62	13.3 (9.0, 20.1)	0.344	1	0.343
Female	25 / 58	11.9 (10.3, 25.7)		0.78 (0.47, 1.31)	
Age at diagnosis, years					
< 65	45 / 86	11.9 (9.7, 19.3)	0.791	1	0.790
≥ 65	15 / 34	11.7 (5.3, UD)		0.92 (0.51, 1.66)	
Smoking history					
Never	42 / 91	14.5 (10.7, 22.1)	0.017	1	0.025
Former / Current	18 / 29	7.9 (3.5, 17.4)		1.94 (1.12, 3.38)	
EGFR mutation type					
Exon 19 deletion	40 / 83	15.0 (10.9, 22.1)	< 0.001	1	0.008
L858R	12 / 27	11.2 (6.5, UD)		1.19 (0.62, 2.28)	
Others	6 / 8	4.5 (1.7, UD)		5.51 (2.23, 13.64)	
Brain metastasis at start of afatinib					
No	40 / 80	15.0 (10.9, 20.6)	0.140	1	0.153
Yes	20 / 40	7.9 (5.1, 13.3)		1.50 (0.87, 2.57)^a^	
Starting dose^b^					
30 mg	23 / 58	10.7 (6.5, UD)	0.105	1	0.113
40 mg	37 / 61	15.0 (10.8, 20.6)		0.63 (0.36, 1.11)	
Amongst patients with no brain metastasis:					
Starting dose^b^					
30 mg	10 / 35	UD	0.897	1	0.898
40 mg	30 / 44	15.0 (10.8, 22.1)		0.95 (0.44, 2.04)	
Amongst patients with brain metastasis:					
Starting dose					
30 mg	13 / 23	5.3 (3.1, 10.7)	0.040	1	0.041
40 mg	7 / 17	13.3 (6.5, UD)		0.39 (0.15, 0.99)	
Amongst patients on 30 mg starting dose:					
Brain metastasis					
No	10 / 35	UD	0.007	1	0.010
Yes	13 / 23	5.3 (3.1, 10.7)		2.96 (1.29, 6.79)	
Amongst patients on 40 mg starting dose:					
Brain metastasis					
No	30 / 44	15.0 (10.8, 22.1)	0.567	1	0.558
Yes	7 / 17	13.3 (6.5, UD)		0.79 (0.34, 1.80)^a^	

*PFS* Progression-free survival, *NA* Not applicable, *UD* Undefined

^a^Non-proportional hazards

^b^One patient had a starting dose of 20 mg. This patient was excluded

### Characteristics of patients with brain metastasis initiated on 30 mg vs. 40 mg

**Fig. 1:**
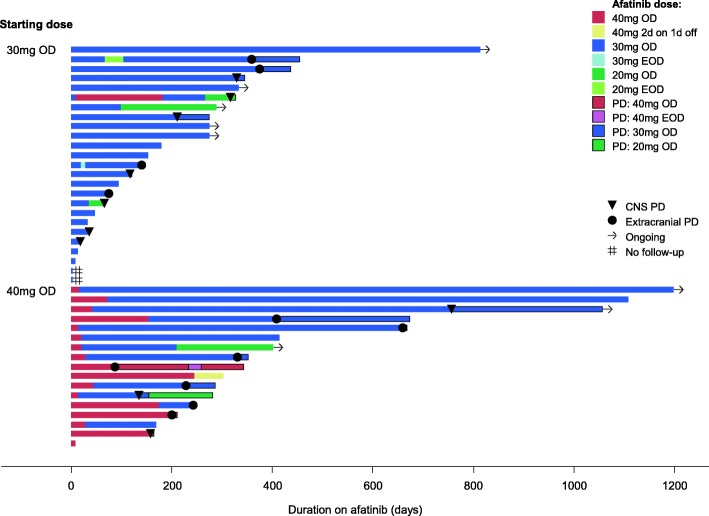
Swimmer plot on dose intensity of afatinib in BM+ patients. Individual swimmer plots for each patient with BM and started on 30 mg OD vs 40 mg OD afatinib, depicting duration and time of intracranial and extracranial disease progression (PD) on different doses of afatinib

We further analyzed the 42 patients with BM prior to afatinib initiation. 25 (59.5%) of them were started on 30 mg afatinib daily and 17 (40.5%) started on 40 mg. There were no significant differences between the 2 groups (40 mg vs 30 mg OD) for important clinical characteristics such as ECOG status, age and smoking history (Table 3). There was greater proportion of females in the 30 mg group (*n* = 16/25, 64.0%) compared to 40 mg group (*n* = 6/11, 35.3%), but the difference was not statistically significant (*p* = 0.067). Of the 42 BM+ patients, 26 had upfront cranial irradiation due to symptomatic or multiple BM with mass effect. Patients who started on 40 mg were more likely to have undergone whole brain radiotherapy (WBRT) prior to afatinib compared to those started on 30 mg (*n* = 14/17, 82.4% vs *n* = 12/25, 48%, *p* = 0.024) for symptomatic BM (Table 3). However, on further analysis to explore the effects of WBRT pre-afatinib, we found that starting dose remained significantly associated with PFS amongst patients who had cranial irradiation pre-afatinib, and in multivariable analysis adjusting for WBRT (Table 4). At time of PD, most patients who started on 30 mg were still on the same dose (81.8%), whereas most of the 40 mg patients had dose reductions (70%) (Fig. 1).

**Table 3:** Comparison of characteristics between BM+ patients on 30 mg and 40 mg starting dose. Comparing the clinical characteristics of patients with brain metastases who started on 30 mg OD vs 40 mg OD of afatinib

Characteristic	Starting dose 30 mg, n (%)	Starting dose 40 mg, n (%)	*p*-value
Age at diagnosis, years			
Median (range)	62 (47–78)	58 (26–76)	0.299
< 65	15 (60.0)	12 (70.6)	0.482
≥ 65	10 (40.0)	5 (29.4)	
Sex			
Female	16 (64.0)	6 (35.3)	0.067
Male	9 (36.0)	11 (64.7)	
ECOG at start of afatinib			
0–1	20 (80.0)	14 (82.4)	1.000
2–3	5 (20.0)	3 (17.6)	
Smoking history			
Never	18 (72.0)	13 (76.5)	1.000
Former/Current	7 (28.0)	4 (23.5)	
Brain RT pre-afatinib			
Yes	12 (48.0)	14 (82.4)	0.024
No	13 (52.0)	3 (17.6)	
Brain RT post-afatinib			
Yes	4 (16.0)	3 (17.6)	1.000
No	21 (84.0)	14 (82.4)	
EGFR mutation type			
Exon 19 deletion	15 (62.5)	9 (52.9)	0.019
Exon 20 insertion	1 (4.2)	0	
Exon 21 L858R	3 (12.5)	8 (47.1)	
Double mutation	5 (20.8)	0	
Unknown	1	0	
Site of progression^a^			
CNS	7 (63.6)	3 (30.0)	0.198
Systemic	4 (36.4)	7 (70.0)	
No PD / unknown:			
Still on afatinib	5	2	
Went on 2nd line	3	4	
No scans / no PD recorded	4	1	
FU at other hospital	2	0	
Afatinib dose at PD, mg			
20	2 (18.2)	1 (10.0)	0.270
30	9 (81.8)	6 (60.0)	
40	0	3 (30.0)	
No PD / unknown	14	7	

Note: Unknown data were not included in the calculation of percentages and p-values

^a^CNS PD: brain. Systemic PD: lung, bone/spine, liver, mediastinal LN, malignant pericardial effusion, nodes, pleura

^b^Note that there were 9 patients (5 on 30 mg and 4 on 40 mg) who were still on afatinib at data cut-off. Dose intensity was calculated up to last follow-up date for these patient

**Table 4:** Multivariable model of afatinib starting dose and WBRT pre afatinib on PFS in BM+ patients at start of afatinib. The relationship between starting dose, WBRT pre-afatinib and PFS in patients with BM shown in a multivariable model

Multivariable analysis	Hazard ratio (95% CI)	*p*-value
Brain RT pre-afatinib		
No	1	
Yes	2.79 (0.93, 8.35)	0.062
Starting dose		
30 mg	1	
40 mg	0.22 (0.07, 0.67)	0.006

### Influence of starting dose on outcomes in patients with brain metastases

**Fig. 2:**
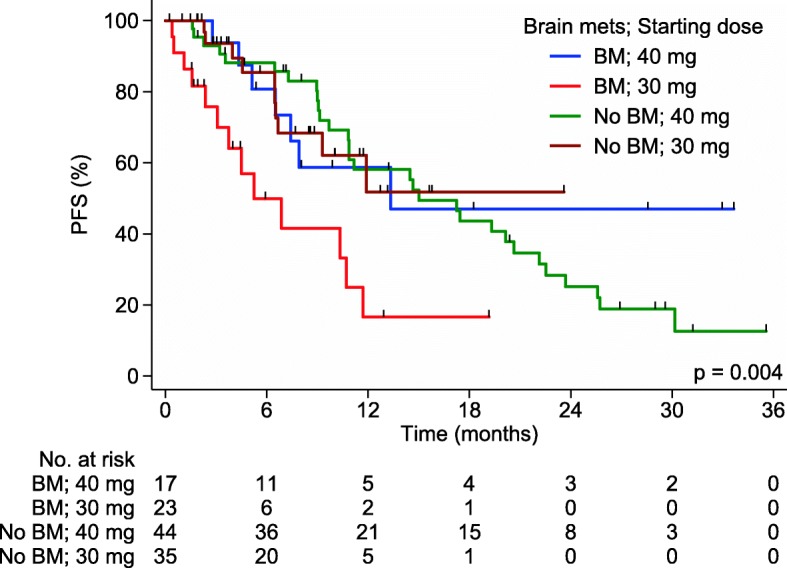
Kaplan-Meier (KM) plot of PFS showing the interaction between BM and starting dose of afatinib. KM plot showing interaction between presence of BM at start of treatment and starting dose of afatinib in our cohort

We next formally explored the interaction between BM and afatinib starting dose (Table 5 and Fig. 2). Amongst patients with BM, median PFS for those who received starting dose 40 mg OD vs. 30 mg OD was 13.3 vs. 5.3 months (HR 0.39, 95% CI 0.15–0.99) (Table 2). However, for patients with no BM at start of afatinib, 40 mg starting dose had no significant impact on median PFS compared to 30 mg (HR 0.95, 95% CI 0.44–2.04). 21/42 BM+ patients had documented PD on afatinib and 1 patient (30 mg group) had both CNS and extracranial/systemic progression at time of PD. For site of first progression, patients who started on 40 mg were less likely to have CNS progression than those on 30 mg (30% vs 63.6%, *p* = 0.198) (Fig. 1), although this was not statistically significant due to the small numbers. Of note, patients with BM who started on 40 mg had similar PFS to patients with no BM (13.3 months vs. 15.0 months; HR 0.79, 95% CI 0.34–1.80). Similar results were obtained when this analysis was repeated in the subset of never-smokers with exon 19 deletions or L858R mutations (Table 5).

**Table 5:** Interaction between brain metastasis and afatinib starting dose in PFS. The interaction effect of brain metastasis and starting dose of afatinib (40 mg vs 30 mg OD) in PFS of patients shown in a multivariable model

	No. of events / patients	Hazard ratio (95% CI)	*p*-value
Brain metastasis; starting dose	60 / 119		
Brain mets; 40 mg		1	
Brain mets; 30 mg		3.73 (1.45, 9.61)	0.006
No brain mets; 40 mg		1.29 (0.57, 2.96)	0.542
No brain mets; 30 mg		1.21 (0.45, 3.23)	0.711
		*p-value of brain mets-starting dose interaction:*	*0.020*
Amongst never smokers with exon 19 deletion or L858R mutation:			
Brain metastasis; starting dose	37 / 84		
Brain mets; 40 mg		1	
Brain mets; 30 mg		5.23 (1.42, 19.28)	0.013
No brain mets; 40 mg		1.67 (0.57, 4.87)	0.345
No brain mets; 30 mg		1.10 (0.29, 4.20)	0.884
		*p-value of brain mets-starting dose interaction:*	*0.011*

## Discussion

In this retrospective analysis, we demonstrated clinical efficacy of afatinib in patients with EGFRm+ NSCLC consistent with large-scale randomized trials [12, 13], with worse outcome in patients with prior smoking histories. However, we did not identify the presence of BM as a negative prognostic factor, prompting us to further examine the patient characteristics and dosing profiles. Interestingly, we found that BM+ patients who commenced on afatinib 40 mg OD had better outcomes than those started on 30 mg OD (median PFS 5.3 vs 13.3 months, *p* = 0.041), and comparable to that of patients without BM (Fig. 2).

While first-line afatinib starting dose of 30 mg OD has been previously reported to have similar clinical efficacy as 40 mg OD and better tolerated in patients with EGFRm+ NSCLC [14], the effect of starting dose on BM has not been studied. In the post-hoc analyses of LUX-Lung 3 and 6 trials reported by Yang and colleagues [7], PFS of patients on afatinib reduced to 30 mg/day due to adverse events was found to be similar to those remaining on 40 mg/day. Although the authors concluded that dose adjustment of afatinib improved incidence of adverse events without compromising on therapeutic efficacy, such effect of afatinib dosing was not examined specifically in the subset of patients with brain metastases. Whereas in our study, we had demonstrated that significant effect of afatinib loading dose (40 mg vs 30 mg OD) on PFS was present only in patients with baseline brain metastases, and not amongst those without brain metastases prior to afatinib initiation – a provocative finding suggesting afatinib dose effect on BM. To the best of our knowledge, this study is the first to demonstrate a difference on outcomes of BM+ patients with different starting doses of afatinib.

Conventionally, WBRT is considered the standard treatment for BM, especially for multiple and symptomatic BM. Although BM+ patients in the 40 mg group were more likely to have undergone WBRT prior to afatinib initiation as compared to the 30 mg group, it is noteworthy that starting dose remained significantly associated with PFS amongst patients who had WBRT before commencing afatinib, and also in multivariable analysis controlling for effect of WBRT. Moreover, patients who started on 40 mg tended to be less likely to progress intracranially than those on 30 mg dose, although not statistically significant due to small numbers. This effect was observed despite the frequency of dose reductions observed, and potentially represents how initial afatinib dose may impact on CNS control in these patients. This corroborates the findings of a competing risk analysis for progression of the LUX-Lung 3, 6, and 7 trials, that the hazard ratio for development of brain metastases as a site of progression was lower for afatinib compared to the control arms, providing another separate validation of the efficacy of afatinib as a brain-penetrant EGFR TKI [15].

The benefit of dose of afatinib on CNS metastases may be driven by the peak plasma concentrations attained, with initial phase I studies showing significant difference in C_max_ (the maximum concentration of drug achieved after administration) when comparing 40 vs 30 mg [16]. In a small case series, Hochmair et al. reported in patients with multiple, symptomatic BM who declined WBRT, afatinib alone could achieve complete intracranial remission [17]. Two other studies also demonstrated effective CNS penetrance of afatinib – a Japanese one with cerebrospinal fluid (CSF) pharmacokinetic data with first-line afatinib treatment [18], and another German series demonstrating CNS activity in patients with BM progressing on first-generation TKIs [6]. Additional studies directed at overcoming CNS treatment failure include high-dose gefitinib and erlotinib given in a pulsatile manner, highlighting the importance of C_max_ on intracranial responses [19, 20].

The main limitations of the current study include the small sample size and retrospective nature of the study, challenging the ability to draw definitive conclusions particularly with regards to afatinib dose effect on patterns of disease progression in BM+ patients. Notwithstanding this, our findings highlight the potential importance of C_max_ in control of brain metastases. This has significant implications on future studies in oncogene-driven NSCLC, where CNS metastases are a common reason for treatment failure and optimal CNS control remains an unmet need. A phase 1b study recently demonstrated the feasibility and tolerability of high-dose intermittent (HDI) afatinib (3 days every 14 days) achieving high plasma concentrations of afatinib, but focused on heavily pretreated advanced T790 M+ NSCLC [21]. Albeit modest activity (7.7%) with HDI afatinib, this may be a potential strategy for patients with CNS metastases. To this end, we have initiated a prospective dose-finding study of continuous (40 mg OD) vs. intermittent high-dose (HDI) afatinib (160 mg × 3 days every 2 weeks) on CNS metastases and leptomeningeal disease in patients with advanced refractory EGFRm+ NSCLC (NCT03711422) to address control of CNS metastases. In this prospective study we will also be assessing the plasma and CSF drug ratios from the 2 different dosing schedules to determine pharmacokinetic efficacy of HDI afatinib on CNS control. Future prospective studies exploring alternative TKI dosing schedules such as intermittent dosing with 40 mg OD, so as to maintain C_max_ while circumventing toxicities from continuous dosing of afatinib, are warranted to specifically address the impact of drug exposure on durability of CNS disease control.

## Conclusion

We demonstrated that in advanced EGFR-mutant NSCLC patients with brain metastases, starting dose of afatinib at 40 mg/day led to better clinical outcomes compared to those who had reduced starting dose of 30 mg/day, possibly due to effects of a higher C_max_ on CNS control. These results also lend support to the CNS activity from afatinib. Moving forward, further elucidation and validation of afatinib dose effect specifically on BM control with concomitant plasma C_max_ testing in a larger prospective study will certainly be crucial.

## Abbreviations

BM: Brain metastases
C_max_: Maximum concentration of drug
CNS: Central nervous system
CR: Complete response
CSF: Cerebrospinal fluid
EGFR: Epidermal growth factor receptor
EGFRm+: EGFR-mutant
HER: Pan-human epidermal growth factor receptor
NSCLC: Non-small cell lung cancer
OD: Once daily
PD: Progressive disease
PFS: Progression free survival
RR: Response rate
TKI: Tyrosine-kinase inhibitor
WBRT: Whole brain radiotherapy
