# Correction to: Influence of afatinib dose on outcomes of advanced EGFR-mutant NSCLC patients with brain metastases

**DOI:** 10.1186/s12885-018-5215-7

**Published:** 2018-12-24

**Authors:** Wan-Ling Tan, Quan Sing Ng, Cindy Lim, Eng Huat Tan, Chee Keong Toh, Mei-Kim Ang, Ravindran Kanesvaran, Amit Jain, Daniel S. W. Tan, Darren Wan-Teck Lim

**Affiliations:** 1 Division of Medical Oncology, National Cancer Centre Singapore, Singapore, Singapore; 2 Clinical Trials & Epidemiological Sciences, National Cancer Centre Singapore, Singapore, Singapore; 3 Genome Institute of Singapore, A*STAR, Singapore, Singapore; 4 Institute of Molecular and Cell Biology, A*STAR, Singapore, Singapore

**Correction to: BMC Cancer (2018) 18:1198**

**DOI: 10.1186/s12885-018-5110-2**

Following publication of the original article [1], the authors notified us of a typographical error in Table 1.

**Table 1:** Patient Baseline Characteristics. The baseline demographics and clinical characteristics of patients with advanced EGFRm + NSCLC treated with first-line afatinib (*n* = 125) in our cohort

Characteristic	No.	%
Sex		
Male	64	51.2
Female	61	48.8
Age at diagnosis, years		
Median	62	
Range	26–86	
Ethnicity		
Chinese	100	80.0
Malay	14	11.2
Indian	3	2.4
Others	8	6.4
Smoking status		
Never	95	76.0
Former	17	13.6
Current	13	10.4
Histotype – NSCLC		
Adenocarcinoma	121	96.8
Adenosquamous carcinoma	1	0.8
NOS	3	2.4
EGFR mutation type		
Exon 19 deletion^[a]^	87	69.6
Exon 21 L858R	27	21.6
Others^[b]^	11	8.8
Brain metastases at baseline		
No	82	65.6
Yes	42	33.6
Unknown	1	0.8
Starting dose of afatinib once daily (OD)		
40 mg	62	49.6
30 mg	61	48.8
20 mg	1	0.8
Unknown	1	0.8

^a^E746_A750del; E746_A750delinsIP; E746_A750delinsQP; E746_A750delinsVP; E746_T751delinsV; E746_S752delinsV; E746_P753delinsVS; L747_A750delinsP; L747_T751del; L747_P753delinsS; NOS

^b^E697Q; A763_Y764insFQEA; Double mutation; Unknown NSCLC Non-small cell lung cancer, NOS Not otherwise specified

The corrected Table 1 is presented below.
